# pH Dependence of the Number of Discrete Conformers of Carbonic Anhydrase 2, as Evaluated from Collision Cross-Section Using Ion Mobility Coupled with Electrospray Ionization

**DOI:** 10.5702/massspectrometry.A0064

**Published:** 2018-03-01

**Authors:** Yoshiaki Nabuchi, Kenji Hirose, Mitsuo Takayama

**Affiliations:** 1 Graduate School of Nanobioscience, Yokohama City University, 22–2 Seto, Kanazawa-ku, Yokohama 236–0027, Japan; 2 Biopharmaceutical Market Development, Waters Corporation Asia Pacific Headquarter, 5–14–10 Nishinakanoshima, Yodogawa-ku, Osaka 532–0011, Japan

**Keywords:** IMS, ESI, carbonic anhydrase 2, conformer, CCS

## Abstract

Ion mobility experiments coupled with electrospray ionization (ESI) were conducted to evaluate the folding states of bovine carbonic anhydrase 2 (CA2) under three different pH conditions. Collision cross-section (CCS) of the CA2 ions generated by ESI revealed the presence of six discrete conformers in the gas phase under the conditions employed in this study. The CCS of the most extended conformer was three times larger than that of the most compact one. The charge state distribution of the CA2 ions was indicative of three conformers being present. Although there was consistency in conformer assignment conducted by CCS and charge state distribution, the CCS measurement was shown to be more effective because the information obtained provided more detailed knowledge of the conformation of the protein.

## INTRODUCTION

One of the commonly accepted methods of analyzing protein folding states by mass spectrometry (MS) is to monitor the shift in charge state distribution of multiply-protonated protein molecules, [M+*n*H]*^n^*^+^, generated by electrospray ionization (ESI),^1–7)^ because ESI can directly transfer those ions from the solution to the gas phase. Although the sample solution passes through an electrospray needle with a high electric field, it is thought that analytes remain in solution until the final ionization step.^8)^ If a protein is in a folded conformation, ESI produces gaseous ions with a relatively small charge number, because compactly folded polypeptide chains only allow protonation at the basic side chains that are exposed at the protein surface during the ionization process. In contrast, for an unfolded protein, a significantly large number of protons on the polypeptide chain are accessible, and the ions are shifted to a lower *m*/*z* region.^2–4)^ A multimodal charge state distribution suggests the coexistence of several conformers.^1–5)^

On the other hand, ion mobility spectrometry (IMS) coupled with ESI is now recognized as a powerful method for analyzing protein conformations in the gas phase, because IMS can separate ions having the same *m*/*z* values but with different shapes or sizes. The drift time measured for an ion can be converted into a corresponding collision cross-section (CCS), and the CCS provides information related to the conformation of the protein.^9–16)^

We have evaluated the folding states of bovine carbonic anhydrase (CA2) in the gas phase. CA2 is a metalloprotein that catalyzes the reversible hydration of CO_2_, in other words, CO_2_+H_2_O↔HCO_3_^−^+H^+^.^17–19)^ CA2 consists of 259 amino acids, in which a Zn^2+^ ion is bound to the active center by three histidine-imidazoles and a H_2_O,^17)^ and is involved in various physiological functions, such as respiration, pH regulation, CO_2_ and HCO_3_^−^ transport, and bio mineralization.^20,21)^ We previously reported on the effects of solution pH on the charge state distribution of CA2 ions produced by ESI and demonstrated that monitoring the *m*/*z* change caused by the removal of the Zn^2+^ it was possible to observe the conversion from holo-CA2 to the apo-form.^22)^ From the product ion spectra obtained under several different solvent conditions, we showed that the folding states of the C-terminal region of the protein were influenced by the solvent.^23,24)^ However, the relationship between the folding states of CA2 ions and their molecular size have not yet been examined.

In the present study, we used a mass spectrometer equipped with a traveling wave ion mobility system to measure the mobility of multiply-charged CA2 molecules generated by ESI at three different pH conditions. The pH dependence of the number of conformers and the CCS values observed for each charge state was evaluated, and the folding states of the CA2 ions were elucidated from the CCS obtained at different pH conditions. The results were compared to the folding states evaluated from the charge state distribution.

## EXPERIMENTAL

### Materials

CA2 from bovine erythrocytes was purchased from Sigma (St. Louis, MO). Equine myoglobin and equine cytochrome *c*, which were used as calibrants for CCS measurements, were obtained from Sigma. All other reagents, such as acetic acid, ammonium acetate, and methanol were of the highest grade available and obtained from Wako Pure Chemical Industries, Ltd. (Osaka, Japan). Purified water was prepared by Milli-Q Advantage A10 (Merck Millipore, Billerica, MA).

### Sample preparation

An approximately 100 pmol/μL solution of CA2 was prepared by dissolving the protein in purified water. To dilute the CA2 solution for MS, the following solvents were prepared: (1) 0.1% formic acid (pH 2.6), (2) 0.3% acetic acid (pH 3.8), and (3) 20 mM ammonium acetate solution (pH 6.5). The pH of these solutions was adjusted with aqueous ammonia. The analyte CA2 concentration prepared to acquire ESI mass spectra was approximately 10 pmol/μL. The CA2 solutions were maintained at ambient temperature for at least 15 min to complete the conformational shift and the samples were then infused into a mass spectrometer.

### Mass spectrometry

Mass spectra were obtained with a SYNAPT G2-Si HDMS quadrupole IMS orthogonal acceleration time-of-flight mass spectrometer equipped with an electrospray ion source and a MassLynx data processor (Waters Corp., Milford, MA). The mass spectrometer was set to detect positive ions. The following data acquisition parameters were employed: electrocapillary voltage of 3.0 kV, sample cone voltage of 30 V, source temperature of 100°C, and desolvation temperature of 200°C. To measure ion mobility, nitrogen was used as a buffer gas, and IMS cell pressure was maintained at 3.1 mbar. The IMS wave velocity was 500 m/s and the wave pulse height was 35 V. The flow rate at which sample solutions were directly infused was 5.0 μL/min.

The obtained drift times were converted into CCS values using the procedure outlined by Robinson *et al.*^9,25)^ Equine myoglobin and equine cytochrome *c*, each of which were dissolved in 49% (v/v) methanol containing 2% (v/v) acetic acid, were used as calibrants and the calibration data were obtained using the same IMS parameters as those for CA2. Myoglobin ions of 15+ to 24+ and cytochrome *c* ions of 13+ to 19+ were used for the calibration plot. The entire range of CCS was obtained from the formula derived from the calibration plot. The CCS for CA2 was calculated from the published X-ray structure^17)^ (RCSB PDB: 1V9E) using the MOBCAL software.^26,27)^

To estimate the number of independent conformers in ESI mass spectra, chemometric processing was conducted using the Origin 8.5 software (Origin Corp., Northampton, MA) as described previously.^28)^

## RESULTS AND DISCUSSION

### ESI mass spectra of CA2 at widely varying pH conditions

**Figure 1:**
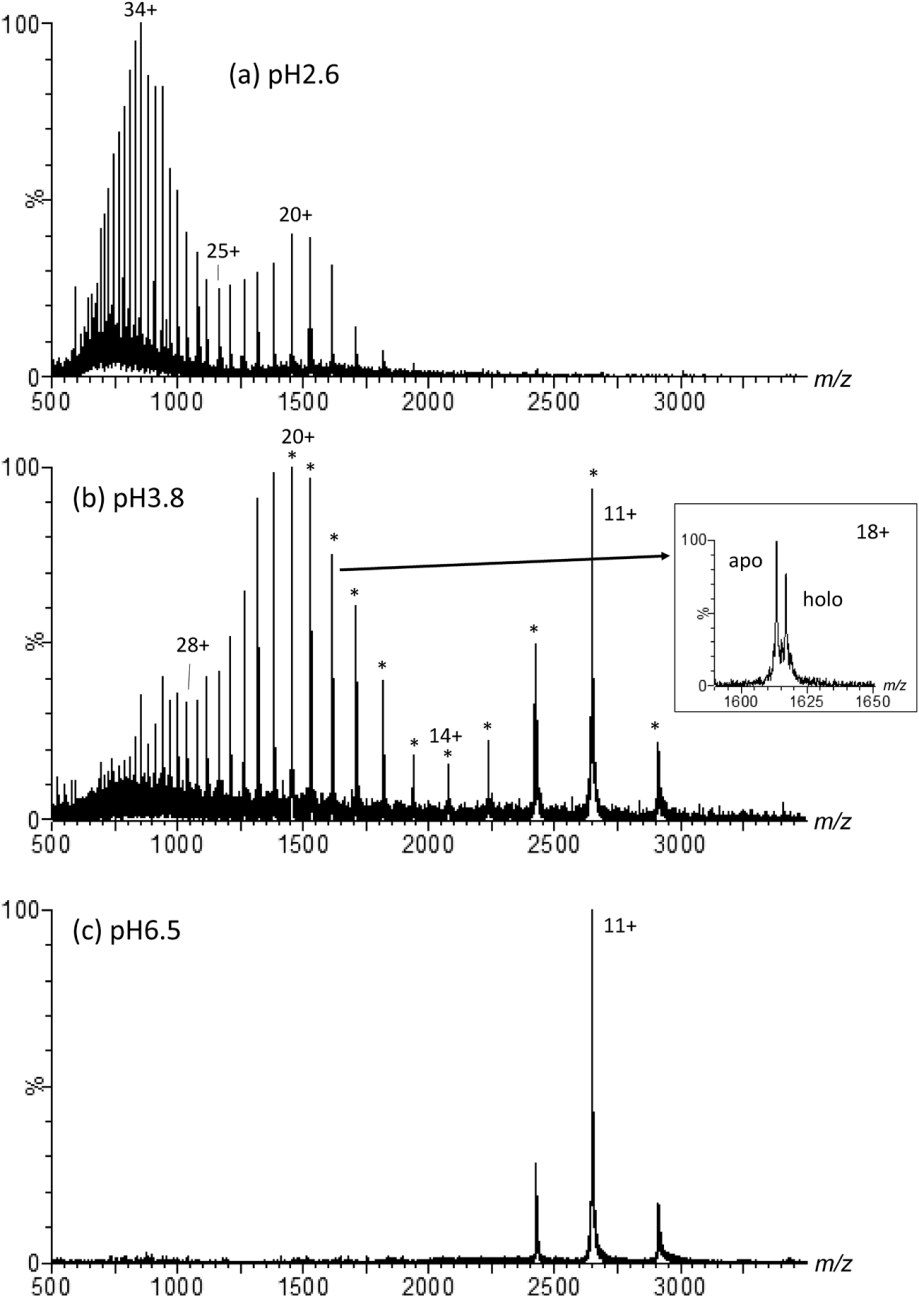
Fig. 1. ESI mass spectra of CA2 measured at three different pH conditions.

ESI mass spectra of CA2 obtained at three different pH conditions are shown in Fig. 1. At pH 2.6, two local maxima at 20+ (*m*/*z* 1452.2) and 34+ (*m*/*z* 854.6) and a local minimum at 25+ (*m*/*z* 1162.0) were observed in a bimodal charge state distribution of [M+*n*H]*^n^*^+^ corresponding to apo-CA2 (Fig. 1a). In the spectrum obtained at pH 3.8, ions corresponding to both apo-CA2 and holo-CA2 were detected. The multiply-charged holo-ions, [M+*n*H+Zn]*^n^*^+^, were observed from 10+ to 20+ and the apo-ions [M+*n*H]*^n^*^+^ were observed at all the charge numbers except 10+. In the spectrum, a trimodal charge state distribution was observed with local minima at 14+ (*m*/*z* 2074.0 for the apo-ion and *m*/*z* 2078.6 for the holo-ion) and 28+ (*m*/*z* 1037.5 for the apo-ion), as shown in Fig. 1b. The distributions of the apo- and holo-ions were not correlated with the trimodal charge state distribution. At pH 6.5, holo-CA2 ions from 10+ to 12+ were observed with the maximum at 11+ (*m*/*z* 2645.2, Fig. 1c).

A CA2 concentration of approximately 10 pmol/μL was used for the measurements. CA2 was dissolved in (a) 0.1% formic acid (pH 2.6), (b) 0.3% acetic acid, adjusted to pH 3.8, (c) 20 mM ammonium acetate (pH 6.5). Bi- and tri-modal charge state distributions were observed at pH 2.6 and 3.8, respectively. The holo-CA2 ions were dominantly observed in pH 6.5. Asterisks indicate ions that are both holo- and apo-CA2. Three ensembles A–C were assigned from the charge state distribution.

**Figure 2:**
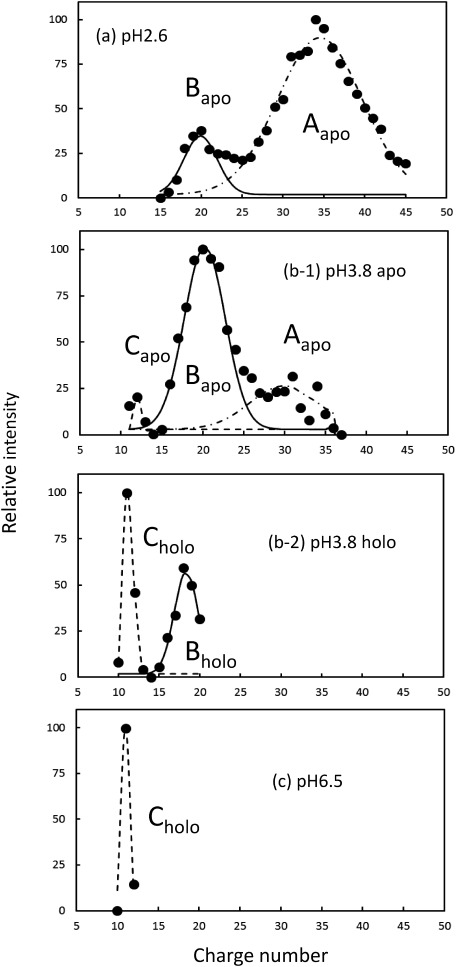
Fig. 2. The results of deconvolution of charge state distribution of CA2 ions in ESI mass spectra obtained at three different pH conditions.

We also conducted a chemometric analysis to determine the number of independent conformers in the multimodal charge state distribution of ESI mass spectra (Fig. 1). The results obtained suggested that CA2 ions were composed of three components for the apo-ions (A–C) and two components for the holo-ions (B and C) under the conditions used in this study (Fig. 2). This indicates that apo- and holo-CA2 consist of three and two conformers, respectively. The enzymatic activity of CA2 is known to be proportional to the binding rate of Zn^2+^, and the enzyme has both hydration and dehydration activities at pH 6.5.^29)^ The CA2 ions observed at pH 6.5 were holo-CA2 ions and belonged to component C. Therefore, component C reflects the folded conformation of CA2, while the ions comprising component A are all in the apo-form. Indicating that the component likely consists of inactive extended conformers. On the other hand, component B was observed at pH 2.6 and pH 3.8 but not at pH 6.5, indicating that ions belonging to this component do not represent physiologically active conformers. At pH 3.8, component B contains some holo-CA2 ions with lower charge numbers. It can be concluded that component B consists of apo- and holo-ions, suggesting that the component might be in a transition state to component A which is dominantly observed at pH 2.6.

The raw data are shown in Fig. 1. Presence of three components was indicated.

### pH dependence of the number of discrete conformers observed in IMS experiments

**Figure 3:**
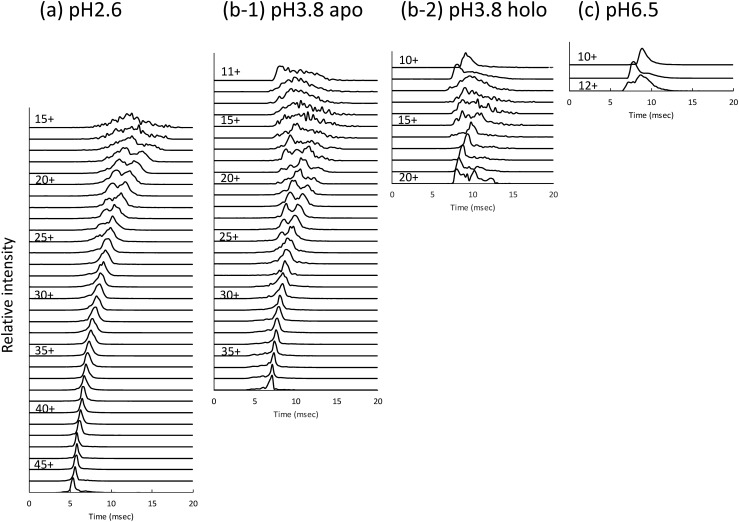
Fig. 3. Driftgrams of multiply-charged CA2 ions (a) at pH 2.6, (b-1) apo-ions at pH 3.8, (b-2) holo-ions at pH 3.8, and (c) at pH 6.5.

Ion mobility experiments were performed to obtain information concerning the gas-phase conformation of the CA2 ions at pH 2.6, 3.8, and 6.5. The driftgrams obtained are shown in Fig. 3. The singlet peak observed in higher charge numbers 29+ to 47+ at pH 2.6 suggests that the ions consist of only one conformer. The peak shape became broader as the charge number decreased, and doublet peaks clearly appeared from 25+ (Fig. 3a). At pH 3.8 for apo-ions (Fig. 3b-1), a singlet peak in the higher charge number ions and doublet peaks from 25+ were observed. The presence of more than two peaks was assumed, since the charge number decreased, suggesting the presence of several conformers. For holo-ions (Fig. 3b-2), the number of the peaks observed in each charge number ion was similar with that of the apo-ions, but the peak shape tended to become narrower than that of apo-ions in the higher charge state. This may be due to a stabilizing effect of Zn^2+^ on the conformation of the molecule.

At pH 6.5 (Fig. 3c), doublet peaks were obtained at 12+ charge state, which were shifted to a singlet peak at 10+. These driftgrams were similar to those of the holo-ion at pH 3.8. Comparing the driftgrams obtained at three different pH conditions, the drift time and peak shape of ions with the same charge number were relatively similar to each other. This suggests that the charge number of the ions is a factor in determining the size of CA2 ions.

### The pH dependence of the CCS on the conformers observed by IMS

**Figure 4:**
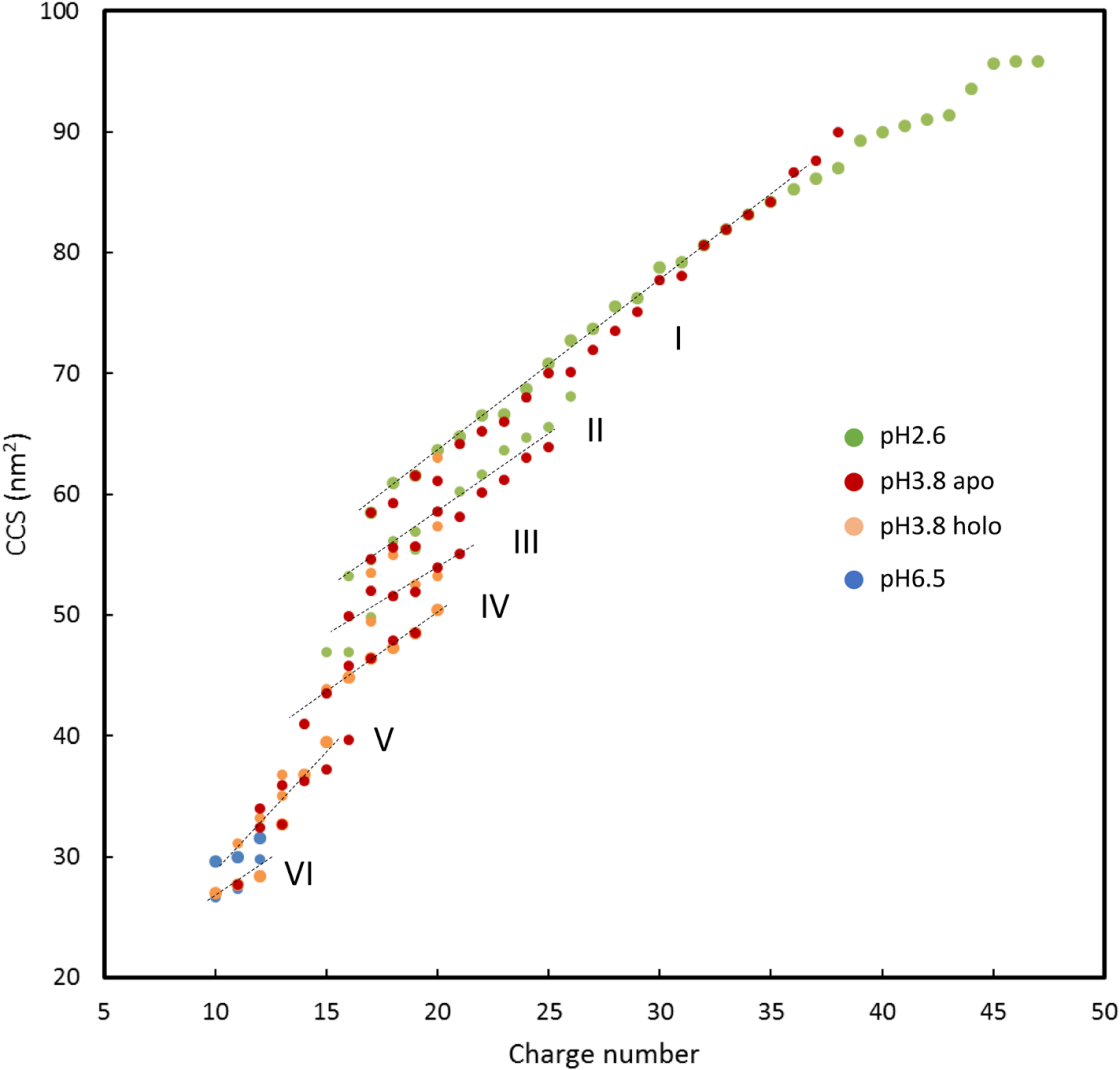
Fig. 4. Plot of charge number *vs.* peak top CCS of driftgrams obtained under the three pH conditions shown in Fig. 3.

The peak top CCS of the driftgrams was obtained for the three pH conditions and the CCS was plotted against the charge number of the ions, as shown in Fig. 4. From the plot, the peak tops could be divided into six groups, indicating the presence of six conformers (I–VI). At pH 2.6, conformers I and II were predominant, while conformers V and VI were predominant at pH 6.5. The remaining conformers III and IV were mainly observed at pH 3.8.

The plots were revealed to be divided into six conformers (I–VI).

**Figure 5:**
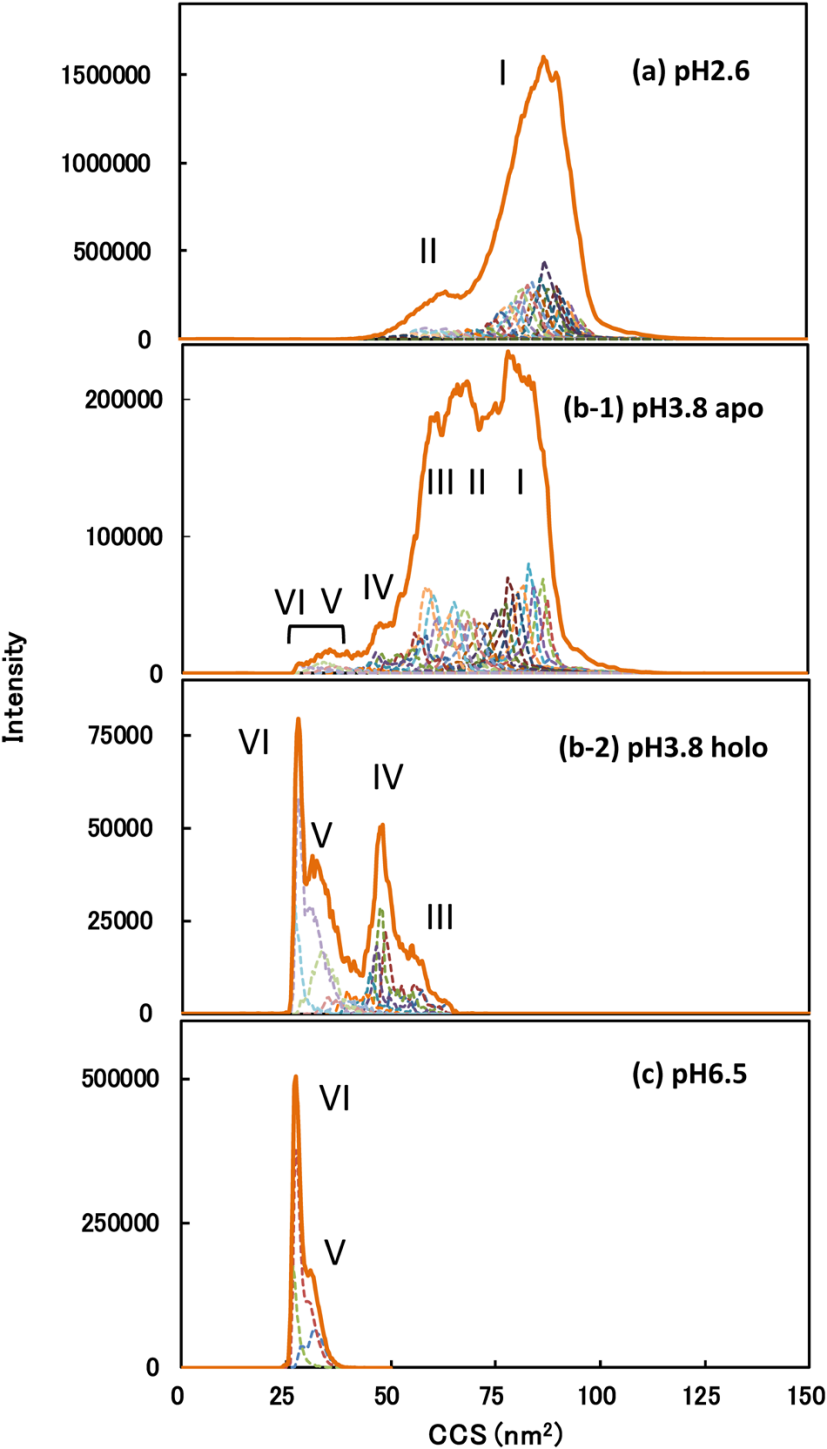
Fig. 5. CCS distribution of CA2 ions produced under three different pH conditions.

The CCS values for the peaks observed on the driftgrams at each charge number were calculated, and the intensity of the ions was plotted against the obtained CCS (dotted line in Fig. 5). The sum total of the intensity of each plot is shown as a solid line in Fig. 5. In the case of the sum total plot at pH 2.6, two conformers I and II corresponding to apo-CA2 were found with CCS values that ranged from 50 to 120 nm^2^ (Fig. 5a), and the top of the peak of conformer I was 87 nm^2^. Both the range of CCS values and peak widths of these conformers were much wider than the other conformers, indicative of the extended shape of conformers I and II. In the case of pH 3.8 apo-ions, the CCS distribution showed a wide range from 25 to 105 nm^2^ and more than 50 nm^2^ ions were abundant. Conformers I, II, and III were contained in this abundant region. In the region less than 50 nm^2^, three conformers corresponding to IV, V, and VI were considered to be present but conformers V and VI were not observed for a specific peak (Fig. 5b-1). In the case of pH 3.8 holo-ions, the CCS distribution showed a wide range from 25 to 65 nm^2^ and the presence of four conformers, III to VI was evident (Fig. 5b-2). A clear valley at 40 nm^2^ between conformers IV and V was observed. This indicates that these conformers may be separated by an energy barrier. The CCS distribution for conformers V and VI was similar to that at pH 6.5 (Fig. 5c), indicating that the folding states of conformers V and VI at pH 3.8 holo are similar to those at pH 6.5. At pH 6.5 (Fig. 5c), ions corresponding to holo-CA2 were revealed to be composed of two conformers, V and VI. The CCS for these two conformers was between 20 and 40 nm^2^, and the peak top for conformer VI was approximately 27 nm^2^. This CCS value is consistent with the value taken from X-ray crystal structure calculated *via* the MOBCAL trajectory method, indicating 24 nm^2^. This suggests that the folding state of conformer VI may be similar with that of the crystal structure. Holo-ions observed at pH 3.8 and 6.5 were distributed in a smaller CCS region than that for apo-ions. This suggests that the coordination of Zn^2+^ may contribute to maintaining the compact shape of the ions.

The dashed lines show the CCS distribution of each multiply-charged ion obtained under the specified condition. The bold line shows the sum total of the dashed lines. The measurements were conducted at pH (a) 2.6, (b) 3.8, and (c) 6.5. The maximal CCS became larger with decreasing pH of the sample solution. Roman numerals indicate the corresponding conformers shown in Fig. 4.

## CONCLUSION

Ion mobility experiments of multiply-charged CA2 ions generated by ESI at three different pH conditions indicated that the gas phase CA2 ions have six discrete conformers (I–VI) with CCS ranging from 20 to 120 nm^2^ under the condition employed in this study. The CCS of peak top of the most compact component VI was 27 nm^2^, whereas that of most extended component I was 87 nm^2^. The CCS of the most compact conformer was consistent with that of the X-ray crystal structure. The conformation of the CA2 ions was analyzed by evaluating the charge state distribution and the results suggest that three components in the apo-ions and two components in the holo-ions are present (A–C, Fig. 2), whereas mobility measurements showed the presence of six conformers (I–VI). The mobility and CCS values reported here suggest that component A included conformer I, component B included conformers II, III, and IV, and component C included conformers V and VI (Figs. 2 and 5). Although there was consistency between the methods, mobility measurements were shown to be more effective because the information obtained gave more detailed information regarding the conformation of the protein.

## Notes

Mass Spectrom (Tokyo) 2018; 7(1): A0064
